# Acute Low-Volume High-Intensity Interval Exercise and Continuous Moderate-Intensity Exercise Elicit a Similar Improvement in 24-h Glycemic Control in Overweight and Obese Adults

**DOI:** 10.3389/fphys.2016.00661

**Published:** 2017-01-09

**Authors:** Lewan Parker, Christopher S. Shaw, Lauren Banting, Itamar Levinger, Karen M. Hill, Andrew J. McAinch, Nigel K. Stepto

**Affiliations:** ^1^Clinical Exercise Science Research Program, Institute of Sport, Exercise and Active Living, College of Sport and Exercise Science, Victoria UniversityMelbourne, VIC, Australia; ^2^Institute for Physical Activity and Nutrition, School of Exercise and Nutrition Sciences, Deakin UniversityGeelong, VIC, Australia; ^3^Clinical Exercise Science Research Program, Institute of Sport, Exercise and Active Living, College of Health and Biomedicine, Victoria UniversityMelbourne, VIC, Australia; ^4^Monash Centre for Health Research and Implementation, School of Public Health and Preventative Medicine, Monash University Clayton VictoriaMelbourne, VIC, Australia

**Keywords:** HIIT, continuous glucose monitoring, PCOS, redox, postprandial, oxidative stress

## Abstract

**Background:** Acute exercise reduces postprandial oxidative stress and glycemia; however, the effects of exercise intensity are unclear. We investigated the effect of acute low-volume high-intensity interval-exercise (LV-HIIE) and continuous moderate-intensity exercise (CMIE) on glycemic control and oxidative stress in overweight and obese, inactive adults.

**Methods:** Twenty-seven adults were randomly allocated to perform a single session of LV-HIIE (9 females, 5 males; age: 30 ± 1 years; BMI: 29 ± 1 kg·m^−2^; mean ± SEM) or CMIE (8 females, 5 males; age: 30 ± 2.0; BMI: 30 ± 2.0) 1 h after consumption of a standard breakfast. Plasma redox status, glucose and insulin were measured. Continuous glucose monitoring (CGM) was conducted during the 24-h period before (rest day) and after exercise (exercise day).

**Results:** Plasma thiobarbituric acid reactive substances (TBARS; 29 ±13%, *p* < 0.01; mean percent change ±90% confidence limit), hydrogen peroxide (44 ± 16%, *p* < 0.01), catalase activity (50 ± 16%, *p* < 0.01), and superoxide dismutase activity (21 ± 6%, *p* < 0.01) significantly increased 1 h after breakfast (prior to exercise) compared to baseline. Exercise significantly decreased postprandial glycaemia in whole blood (−6 ± 5%, *p* < 0.01), irrespective of the exercise protocol. Only CMIE significantly decreased postprandial TBARS (CMIE: −33 ± 8%, *p* < 0.01; LV-HIIE: 11 ± 22%, *p* = 0.34) and hydrogen peroxide (CMIE: −25 ± 15%, *p* = 0.04; LV-HIIE: 7 ± 26%; *p* = 0.37). Acute exercise provided a similar significant improvement in 24-h average glucose levels (−5 ± 2%, *p* < 0.01), hyperglycemic excursions (−37 ± 60%, *p* < 0.01), peak glucose concentrations (−8 ± 4%, *p* < 0.01), and the 2-h postprandial glucose response to dinner (−9 ± 4%, *p* < 0.01), irrespective of the exercise protocol.

**Conclusion:** Despite elevated postprandial oxidative stress compared to CMIE, LV-HIIE is an equally effective exercise mode for improving 24-h glycemic control in overweight and obese adults.

## Introduction

Physical inactivity and obesity are major risk factors for impaired glycemic control, insulin resistance and type 2 diabetes (Valko et al., 2007; Fisher-Wellman et al., 2009). Compared to continuous moderate-intensity exercise (CMIE), high-intensity interval exercise (HIIE) has been shown to elicit comparable and/or greater improvements in glycemic control (Gibala et al., 2012; Liubaoerjijin et al., 2016). Notably, an improvement in glycemic control can be seen even after a single bout of exercise (Gillen et al., 2012; van Dijk et al., 2012; Little et al., 2014). However, current laboratory based techniques used for assessing glycemic control, such as the oral glucose tolerance test and the homeostatic model assessment of insulin resistance (HOMA-IR), may not always reflect functional improvements in glycemic control under free-living conditions (Mikus et al., 2012). Continuous glucose monitoring (CGM) is a reliable and valid method for measuring 24-h glycemic status, glycemic variability, and postprandial responses to meals under free-living conditions (Tubiana-Rufi et al., 2007; Mikus et al., 2012). A single bout of HIIE can improve 24-h glycemic control in obese individuals and patients with type 2 diabetes (Gillen et al., 2012; Little et al., 2014). However, only one study has compared the acute effects of HIIE to CMIE when matched for total workload (Little et al., 2014). Consequently, whether shorter duration, lower-volume HIIE (LV-HIIE) provides similar, or greater benefits in 24-h post-exercise glycemic control compared to the currently recommended exercise mode of CMIE is unknown.

Oxidation-reduction (redox) status is reported to mediate glycemic control in both healthy individuals and those with diabetes (Wright et al., 2006; Tiganis, 2011). Oxidative stress occurs as a result of a redox imbalance in favor of excess reactive oxygen species (ROS). This imbalance can result in oxidative modification to DNA, lipids and proteins, playing both a pathological and physiological role in metabolic health (Valko et al., 2007). Chronic systemic oxidative stress is associated with obesity and physical inactivity, and is linked to the development of insulin resistance and type 2 diabetes (Valko et al., 2007). Paradoxically, acute exercise also induces a transient state of elevated oxidative stress (Fisher-Wellman and Bloomer, 2009), yet improves insulin sensitivity and glycemic control (Gillen et al., 2012; van Dijk et al., 2012; Little et al., 2014). While exercise-induced oxidative stress is deemed beneficial and a necessary requirement for optimal tissue functioning and adaptation to physiological stress (Radak et al., 2013), the effects of exercise-intensity on redox status remain unclear.

Elevated basal and/or postprandial hyperglycemia elicited through excess nutrient intake, physical inactivity, and/or insulin resistance, is reported to increase systemic oxidative stress through mitochondrial membrane electron leak and the formation of advanced glycation end products (AGEs) (Wright et al., 2006; Fisher-Wellman and Neufer, 2012). Postprandial oxidative stress can last for up to 4 h after meal consumption and occurs to a greater extent with larger meals that are higher in lipid content (Tucker et al., 2008; Bloomer et al., 2010; Fisher-Wellman and Bloomer, 2010; Fisher-Wellman and Neufer, 2012; Canale et al., 2014). In contrast to exercise-induced oxidative stress, excess postprandial systemic oxidative stress contributes to metabolic health complications associated with insulin resistance and type 2 diabetes (Wright et al., 2006; Tucker et al., 2008; Fisher-Wellman et al., 2009; Fisher-Wellman and Neufer, 2012). A single session of low to moderate-intensity exercise in healthy males attenuates the postprandial oxidative stress response to a meal ingested 2 h before (Mc Clean et al., 2007) and 24 h after exercise (Takahashi et al., 2015). Furthermore, high-intensity exercise which elicits greater oxidative stress and antioxidant activity compared to low to moderate intensity exercise (Schneider et al., 2005; Fisher-Wellman and Bloomer, 2009; Parker et al., 2014), may also attenuate postprandial oxidative stress (Tyldum et al., 2009; Gabriel et al., 2012). Considering the impact of HIIE on postprandial oxidative stress are equivocal (Canale et al., 2014), and overweight and inactive population's exhibit greater basal and postprandial oxidative stress than healthy controls (Tucker et al., 2008; Fisher-Wellman et al., 2009), further research is warranted.

The aim of this study was to test the hypotheses that LV-HIIE would improve 24-h glycemic control and postprandial redox status in overweight and obese males and females to a greater extent than CMIE.

## Materials and methods

### Participants

Twenty-seven physically inactive males and females, who were on average overweight (BMI range: 21.4–45.0 kg·m^−2^; 23 out of 27 participants had a BMI >25), volunteered to participate in the study. Participant characteristics are reported in Table 1. Females diagnosed with polycystic ovary syndrome (PCOS) were included in the study as they have an intrinsic insulin resistance and are at a 4-fold greater risk of developing type 2 diabetes (Stepto et al., 2013; Cassar et al., 2016). PCOS diagnosis was self-reported, and supported by personal medical records that adhered to the Rotterdam criteria (Fauser et al., 2004). Participants were sedentary and had not participated in any regular moderate to high levels of physical activity within the past 3 months. Exclusion for participation included medications known to affect insulin secretion and/or insulin sensitivity; musculoskeletal or other conditions that prevent daily activity; and symptomatic or uncontrolled metabolic or cardiovascular disease. Women with PCOS taking medication (e.g., metformin) were included if medication was stable (>3 months) and were asked to withdraw medication 48 h prior to, and throughout the experimental phase of the study. Females were tested in the early follicular phase of the menstrual cycle. Verbal and written explanations about the study were provided prior to obtaining written informed consent. This study was approved by the Victoria University Human Research Ethics Committee and carried out in accordance with The Code of Ethics of the World Medical Association (Declaration of Helsinki) for experiments involving humans (World Medical, 2013).

**Table 1:** **Descriptive characteristics of participants in the LV-HIIE and CMIE protocol group**.

	**LV-HIIE**	**CMIE**	***p*-value**
Participants	14	13	
Males	5	5	
Females	9	8	
Females with PCOS	6	5	
Age (years)	30 ± 1	30. ± 2	0.96
Height (cm)	169.5 ± 2.7	166.4 ± 2.3	0.40
Weight	84.1 ± 5.1	83.3 ± 5.6	0.92
BMI (kg·m^−2^)	29.2 ± 1.4	30.0 ± 1.8	0.70
BMI >25 kg·m^−2^	4 males 8 females	4 males 7 females	
Waist to hip ratio	0.82 ± 0.02	0.84 ± 0.03	0.85
Systolic blood pressure (mm Hg)	122 ± 3	117 ± 3	0.20
Diastolic blood pressure (mm Hg)	85 ± 3	78 ± 3	0.07
W_max_ (Watts)	175 ± 19	170 ± 14	0.82
Max heart rate (BPM)	186 ± 3	180 ± 4	0.22
VO_2max_ (ml·kg^−1^·min^−1^)	28.7 ± 2.2	28.8 ± 1.9	0.98
Total exercise session work (kJ)	147 ± 13	191 ± 15^*^	0.04
Total exercise session duration (min)	24	38^*^	<0.01
HOMA2-IR	1.4 ± 0.1	1.4 ± 0.2	0.78
Fasting glucose (mmol/l)	4.5 ± 0.1	5.0 ± 0.3	0.12
Fasting insulin (pmol/l)	88 ± 7	84 ± 12	0.78

*Values are mean ± SEM*.

* *p < 0.05 compared to LV-HIIE. LV-HIIE, low-volume high-intensity interval exercise; CMIE, continuous moderate-intensity exercise; PCOS, polycystic ovary syndrome; HOMA2-IR, homeostatic model assessment of insulin resistance version 2. BMI: body mass index*.

### Study design

**Figure 1:**
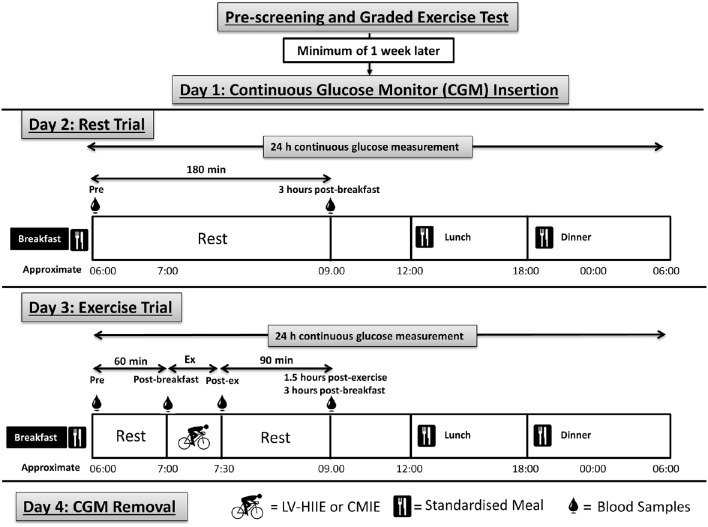
**Detailed schematic of the research methodology and procedures**. Participants were pre-screened and randomized into either LV-HIIE or CMIE exercise groups. Participants than underwent 4 consecutive days of testing. Day 1 consisted of CGM insertion and instruction of its appropriate use. Day 2 consisted of a rest non-exercise day. Day 3 was identical to day 2 with the addition of a single session of either LV-HIIE or CMIE performed 1 h after breakfast consumption. Day 4 consisted of removal of the CGM. Participants were provided a standard breakfast, lunch, dinner and snacks, to eat throughout the 4-day testing period. Meals were consumed at the same time of day over the testing period. Venous blood samples were taken at the time points indicated in the figure. CMIE, Continuous moderate-intensity exercise; LV-HIIE, low-volume high-intensity interval exercise; CGM, continuous glucose monitor.

Participants were instructed to abstain from physical activity, alcohol and caffeine consumption (48 h) prior to, and throughout, the 4 consecutive days of the experimental period. In brief, day one involved insertion of the CGM and participant familiarization; Day two was a rest day with dietary control; Day three was an exercise day (LV-HIIE or CMIE) with dietary control; Day four involved the removal of the CGM (Figure 1).

### Screening and preliminary testing

Participants were pre-screened via a medical history and risk assessment questionnaire. Eligible participants underwent body composition analysis, and a graded exercise test (GXT) on a cycle ergometer (Lode Excalibur Sport) to measure aerobic capacity (VO_2max_) and maximal power output (W_max_). Expired gases were collected and analyzed via a metabolic system (Moxus Modular VO2 System). The GXT protocol consisted of 3 min cycling (60 RPM) at 50 W, increasing by 25 W every 3 min for the first three stages, and then increasing every 1 min thereafter. Participants cycled until they were unable to maintain 50 revolutions per min. The maximum wattage obtained (W_max_) during the exercise test was used to calculate the workload for the LV-HIIE or CMIE in the main experiment. Heart rate (HR) was recorded using a 12-lead electrocardiograph system along with the 6–20 Borg scale rating of perceived exertion (RPE). One to three weeks after completing the pre-screening session participants were randomized into either the LV-HIIE or CMIE exercise interventions stratified by sex and BMI.

## Experimental phase

### Experimental day 1

Participants reported to the laboratory for fitting of the CGM (Guardian® Real-Time, Medtronic, USA) and detailed instructions of its use (Tubiana-Rufi et al., 2007). Calibration of the CGM was performed three times per day (morning, lunchtime, and evening), and performed at least 2 h after the participants last meal. Participants were instructed to consume the food provided and refrain from any physical activity not prescribed by the researchers. Prior to leaving the laboratory, participants were given a standardized meal (dinner) to take home and consume at their normal meal time.

### Experimental day 2

Participants reported to the laboratory in the morning after an overnight fast and consumed a standardized breakfast. Immediately following breakfast participants rested in the laboratory for a total of 3 h. Blood samples were taken at baseline (prior to breakfast) and 3 h after breakfast. Participants were provided standardized meals for lunch, dinner and snacks to take home and eat at their preferred meal times, and were instructed to detail all food consumption and physical activity in the provided log books.

### Experimental day 3

Participants again reported to the laboratory in the morning after an overnight fast. As per the previous rest day, participants consumed the standardized breakfast. One hour later, participants performed a single session of either the LV-HIIE or CMIE protocol. The participants then rested in the laboratory for 90 min. The standardized meals were again provided including the final day's breakfast (day 4), to be consumed at the same time of day as per the previous 2 days. Blood samples were taken at baseline, pre-exercise (1 h after breakfast), and immediately after exercise, 5, 15, 60, and 90 min after exercise (~3 h after breakfast).

### Experimental day 4

Participants arrived in the laboratory ~3 h after consumption of their standard breakfast and the CGM was removed.

### Exercise protocols

The LV-HIIE protocol consisted of a 5-min warm-up at 50% of the participants W_max_ obtained during the GXT. Following the warm-up, participants performed 8 × 1-min cycling bouts at 100% of W_max_ (175 ± 19 W), interspersed with 1-min active recovery periods cycling at 50 W. A 3-min cool down was then performed at 50% of W_max_. The total workout session duration was 24 min. The CMIE session consisted of 38 ± 1 min cycling at 50% of the participants W_max_ (79 ± 9 W). Total work performed on the cycle ergometer during the LV-HIIE and CMIE sessions are reported in Table 1.

### Dietary control

Daily energy and macronutrient intake for the standardized meals were based on sex, height and weight, and consisted of approximately 55% carbohydrate, 30% fat and 15% protein, adhering with the Australian and New Zealand dietary targets (Council, 2014). With the exception of necessary dietary substitutions (vegetarian, halal etc.) breakfast consisted of Kellogg's® Corn Flakes and Kellogg's® All-Bran®, honey and full cream milk; lunch consisted of canned tuna, tomato, lettuce and carrot roll/s; dinner consisted of sausages, cooked white rice, sweet potato, and mixed frozen vegetables; and snacks consisted of a muffin, banana, and yogurt. To ensure consistency throughout the study, participants were instructed to eat breakfast, lunch and dinner at the same time of day over the 4-day experimental period, and were instructed to log all physical activity (time of day, exercise mode, duration and intensity) and food consumed (time of day, type and quantity of food eaten) in the provided log books.

### Blood sampling

Venous blood was collected from an antecubital vein via an intravenous cannula and collection tube and kept patent with 0.9% sterile saline. Blood was collected in appropriate tubes and immediately centrifuged at 3500 rpm for 15 min at 4°C, the plasma was aliquoted and stored at −80°C until analyzed.

### Biochemical analysis

Whole blood lactate and glucose were analyzed using an automated analysis system (YSI 2300 STAT Plus® Glucose & Lactate Analyzer). Plasma insulin levels were determined in duplicate using radioimmunoassay in accordance with the manufacturer's instructions (HI-14K kit, Millipore). Insulin resistance was estimated using the homeostatic model assessment (version 2) for insulin resistance (HOMA2-IR) using the Oxford Diabetes Trials Unit calculator (https://www.dtu.ox.ac.uk/homacalculator; University of Oxford, UK).

### Plasma redox status analysis

Plasma thiobarbituric acid reactive substances (TBARS; Cayman), catalase activity (Cayman), superoxide dismutase activity (SOD; Cayman) and hydrogen peroxide (Amplex UltraRed assay, Molecular Probes) were determined on a spectrophotometer (xMark microplate spectrophotometer, Bio-Rad Laboratories) in duplicate as per the manufacturer's instructions. Intra-assay coefficients of variation were 2, 3, 3, and 2% for TBARS, SOD, Catalase and hydrogen peroxide, respectively. Inter-assay coefficients of variation were 1, 4, 4, and 1%, for TBARS, SOD, catalase and hydrogen peroxide, respectively.

### Continuous glucose monitor analysis

Five-minute glucose values recorded by the CGM over the 4-day intervention were exported. Meal-times were cross-checked with participants' diet log books and data were checked for missing values and/or abnormal readings. The 24-h period prior to the onset of exercise (rest day) and the 24-h period immediately after the onset of exercise (exercise day), were used to compare 24-h CGM determined glycemic control. For consistency, missing data points were handled as per previous publications using CGM technology (Little et al., 2014). Briefly, if less than 3 consecutive 5-min periods were missing the average of the glucose value before and after were inserted. If greater than 3 consecutive 5-min periods were missing over the 24-h period of comparison, then both the rest and exercise days were adjusted to omit these values. Continuous glucose monitoring data was corrupted for 1 participant in the LV-HIIE, and was excluded from the CGM comparisons.

Average glucose values, peak glucose concentration, total area under the curve (AUC) and incremental AUC (iAUC), were calculated for the 24-h rest day and exercise day period, and the 2-h postprandial period following consumption of breakfast, lunch and dinner. The 24-h glycemic variability measurements of the standard deviation (SD) of the mean glycaemia, the mean amplitude of glycemic excursions (MAGE), and the percentage coefficient of variation (% CV), were calculated using the GlyCulator windows software package as previously described (Czerwoniuk et al., 2011). The percentage of time spent with hyperglycemia (above 7 mmol/l) during the 24-h time period was also determined.

### Statistical analysis

Data were checked for normality and analyzed using Predictive Analytics Software (PASW v20, SPSS Inc.). Comparisons of means for the CGM data were examined using a two-factor repeated measures analysis of variance (ANOVA) with trial day (rest or exercise day) as the within-subject factor and group (LV-HIIE or CMIE) as a between-subjects factor. Comparison of multiple means for biochemical analysis, heart rate and RPE, on the exercise day were analyzed using a two-factor repeated measures ANOVA with time as the within-subjects factor and group (LV-HIIE and CMIE) as the between-subjects factor. A three-factor repeated measures ANOVA was conducted to investigate the effect of exercise on postprandial oxidative stress with time (baseline and 3 h postprandial) and day (rest day and exercise day) as within-subject factors and group (LV-HIIE and CMIE) as the between-subjects factor. Significant interaction and main effects were explored using Fisher's LSD *post-hoc* analysis test. All data are reported as mean ± standard error of mean (SEM) and all statistical analysis were conducted at the 95% level of significance (*p* ≤ 0.05). Trends were reported when *p*-values were greater than 0.05 and less than 0.1.

## Results

Measures of fasting glucose, fasting insulin, and HOMA2-IR were not statistically different between participants with and without PCOS, or between PCOS participants in the LV-HIIE and CMIE groups (*p* > 0.05; data not shown), as such data were pooled together for analysis. Baseline measurements for CGM and biochemical data, and physiological data measured in the GXT, were not significantly different (*p* > 0.05) between LV-HIIE and CMIE groups (Table 1).

### Physiological response to exercise

**Figure 2:**
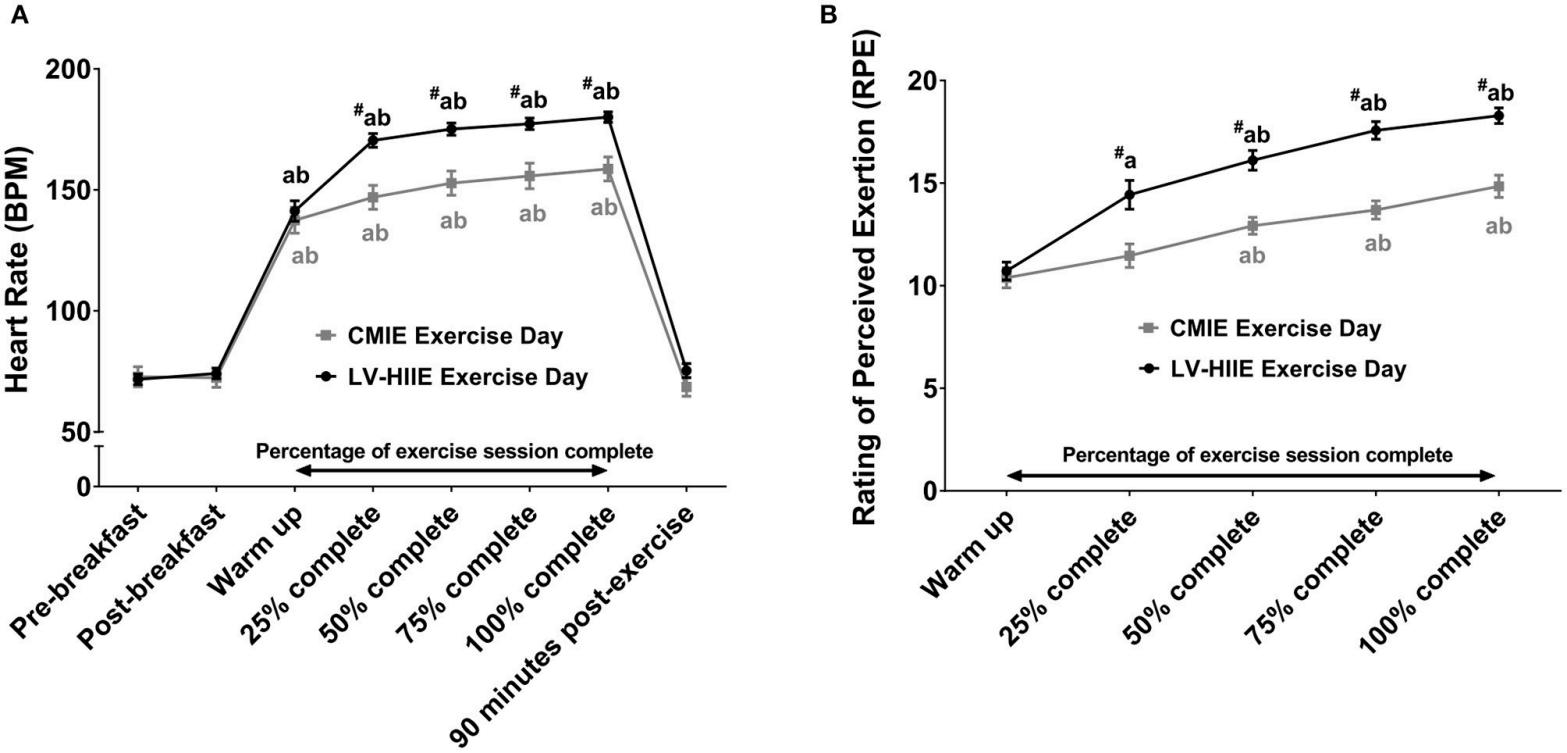
**Rating of perceived exertion and heart rate response to LV-HIIE and CMIE**. Heart rate **(A)** and rating of perceived exertion **(B)** during exercise for both LV-HIIE and CMIE. ^a^*p* < 0.05 compared to pre-breakfast or warm up; ^b^*p* < 0.05 compared to 1-h post-breakfast or repetition 2 (25% of CMIE); ^#^*p* < 0.05 compared to the corresponding time point in the CMIE group. CMIE, continuous moderate intensity exercise; LV-HIIE, low-volume high-intensity interval exercise. Values are means ± SEM.

HR and RPE were significantly higher during LV-HIIE compared to CMIE (Figure 2). Total work (kJ) and exercise duration (minutes) in the CMIE group were significantly greater than LV-HIIE (Table 1).

### Continuous glucose monitor data

**Figure 3:**
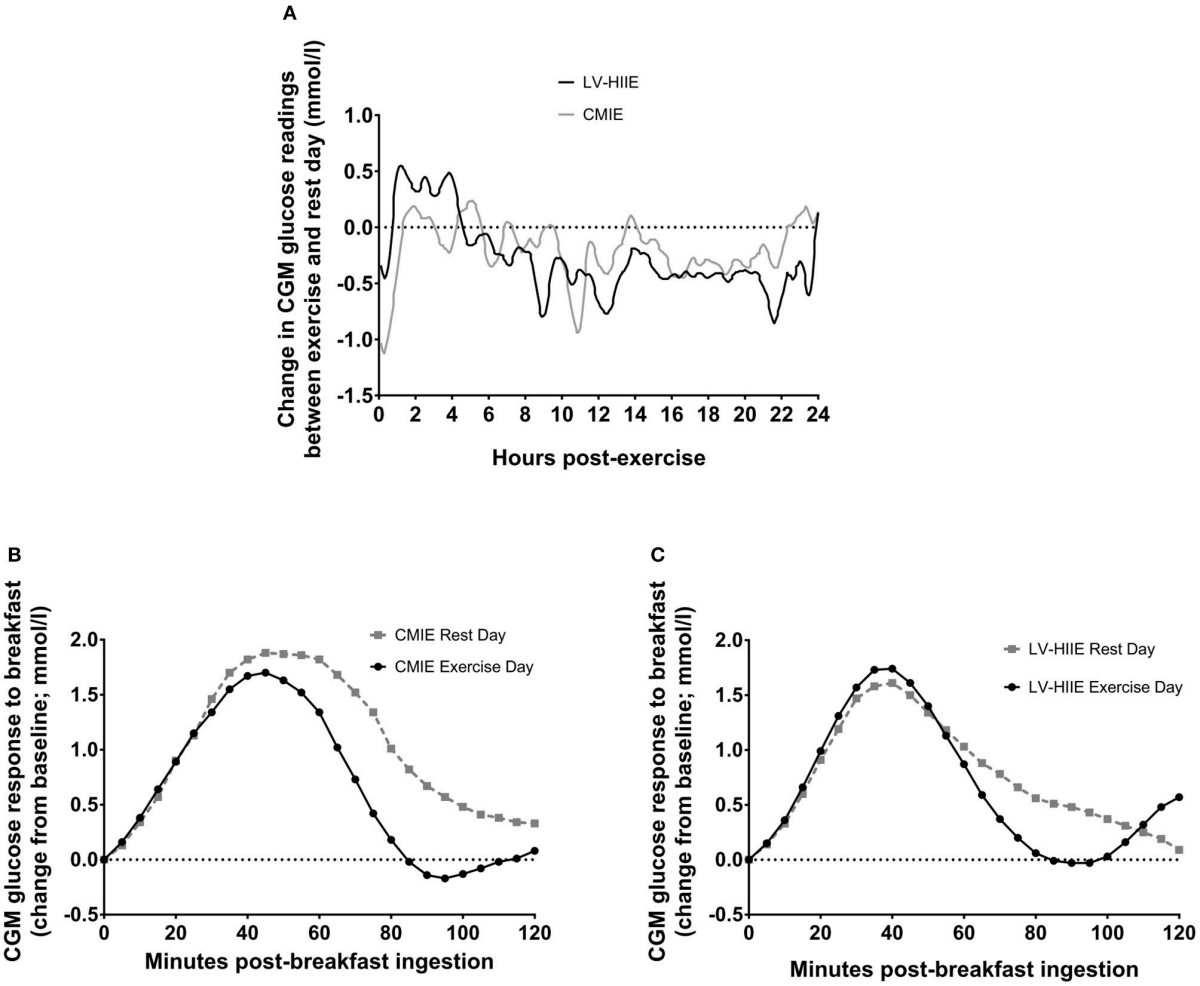
**Continuous glucose monitor traces for 24-h glucose and postprandial breakfast**. Change in average CGM glucose readings (mmol/l) from rest to exercise day over the 24-h period **(A)**. Average postprandial CGM glucose readings (change from baseline; mmol/l) on the rest day and when CMIE **(B)** or LV-HIIE **(C)** were performed 1 h after breakfast consumption. CMIE, continuous moderate intensity exercise; LV-HIIE, low-volume high-intensity interval exercise; CGM, continuous glucose monitor.

Average glucose concentration, glucose AUC, peak glucose concentration, and the percentage of the day spent with glucose values above 7 mmol/l were significantly lower during the 24-h period after exercise compared to the rest day (all *p* < 0.05) with no differences between LV-HIIE and CMIE (all *p* > 0.05; Table 2). Exercise significantly decreased dinner 2-h average glucose concentration, peak glucose concentration, AUC and iAUC (all *p* < 0.05; Table 2). Exercise significantly decreased breakfast total and iAUC (both *p* < 0.05), whereas a significant interaction effect (*p* = 0.04) and subsequent *post-hoc* analysis indicated that only CMIE decreased breakfast 2-h average glucose concentration (*p* < 0.01; Table 2). The glycemic variability measurements of MAGE, SD, and % CV were not significantly different between trial days or groups (all *p* > 0.05; Table 2). Representative graphs of the mean difference between the rest day and exercise day over the 24-h period, and the CGM postprandial breakfast response, are reported in Figure 3.

**Table 2:** **Analysis of continuous glucose monitoring measurements during the rest and exercise day for both LV-HIIE and CMIE sessions**.

**Variable**	**LV-HIIE**		**CMIE**	
	**Rest day**	**Exercise day**	**Rest day**	**Exercise day**
**24 h MEASUREMENT (mmol/l)**				
Average blood glucose	4.7 ± 0.1	4.5 ± 0.1^*^	5.3 ± 0.3	5.1 ± 0.3^*^
Total AUC	6438 ± 192	6077 ± 214^*^	7174 ± 506	6847 ± 475^*^
Peak glucose concentration	7.2 ± 0.3	6.5 ± 0.3^*^	7.9 ± 0.6	7.2 ± 2.0^*^
**2 h PPG (mmol/l)**				
Breakfast	5.1 ± 0.2	5.3 ± 0.3	6.1 ± 0.5	5.4 ± 0.4^*^
Breakfast (1st h)	5.4 ± 0.2	5.4 ± 0.2	6.2 ± 0.4	5.8 ± 0.4
Breakfast (2nd h)	4.9 ± 0.2	4.5 ± 0.2^*^	5.9 ± 0.6	5.0 ± 0.4^*^
Lunch	5.0 ± 0.2	5.1 ± 0.1	5.9 ± 0.3	5.7 ± 0.3
Dinner	5.2 ± 0.3	4.6 ± 0.2^*^	6.2 ± 0.4	5.7 ± 0.3^*^
**2 h PPP (mmol/l)**				
Breakfast	6.1 ± 0.2	6.3 ± 0.3	7.4 ± 0.7	6.8 ± 0.6
Lunch	5.7 ± 0.2	5.9 ± 0.3	7.1 ± 0.4	6.8 ± 0.5
Dinner	5.9 ± 0.4	5.2 ± 0.3^*^	7.1 ± 0.5	6.5 ± 0.4^*^
**2 h iPPP (mmol/l)**				
Breakfast	1.6 ± 0.2	1.7 ± 0.3	2.4 ± 0.4	1.9 ± 0.3
Lunch	1.5 ± 0.2	1.6 ± 0.3	2.5 ± 0.3	2.0 ± 0.4
Dinner	1.5 ± 0.3	1.1 ± 0.2^*^	2.1 ± 0.3	1.8 ± 0.3^*^
**2 h AUC (mmol/l**·**2 h)**				
Breakfast	617 ± 25	609 ± 29^*^	730 ± 63	655 ± 49^*^
Lunch	601 ± 25	610 ± 16	711 ± 36	709 ± 32
Dinner	629 ± 34	569 ± 23^*^	748 ± 53	687 ± 41^*^
**2 h iAUC (mmol/l**·**2 h)**				
Breakfast	88.6 ± 11.9	80.5 ± 18.6^*^	105.0 ± 13.7	71.3 ± 14.0^*^
Lunch	107.7 ± 16.2	93.3 ± 11.7	173.1 ± 20.8	153 ± 27.7
Dinner	107.2 ± 18.0	75.9 ± 20.4^*^	154.3 ± 30	129.4 ± 26.9^*^
**GLYCEMIC VARIABILITY**				
MAGE	2.2 ± 0.2	2.2 ± 0.2	2.6 ± 0.3	2.2 ± 0.3
SD	0.7 ± 0.1	0.7 ± 0.1	0.8 ± 0.1	0.8 ± 0.1
%CV	15.1 ± 1.2	15.7 ± 1.6	15.58 ± 1.3	14.58 ± 1.7
% of 24 h day at >7 mmol/l^δ^	2.7 ± 0.9	0.2 ± 0.2^*^	11.4 ± 6.3	8.5 ± 5.3^*^

*Values are mean ± SEM (n = 13 in both LV-HIIE and CMIE groups for CGM data)*.

* *p < 0.05 are significantly different to the rest day. δ-7 out of 13 participants in the LV-HIIE group and 10 out of 13 in the CMIE group exhibited glucose values higher than 7 mmol/L. LV-HIIE, low-volume high-intensity interval exercise; CMIE, continuous moderate-intensity exercise; MAGE, 24-h mean amplitude of glycemic excursion; SD, 24-h glycemic standard deviation. %CV, 24-h glucose percentage coefficient of variation; PPG, postprandial glucose; PPP, peak postprandial glucose; iPPP, incremental peak postprandial glucose; AUC, area under the glucose curve; iAUC, incremental area under the glucose curve*.

### Exercise day biochemical analysis

**Figure 4:**
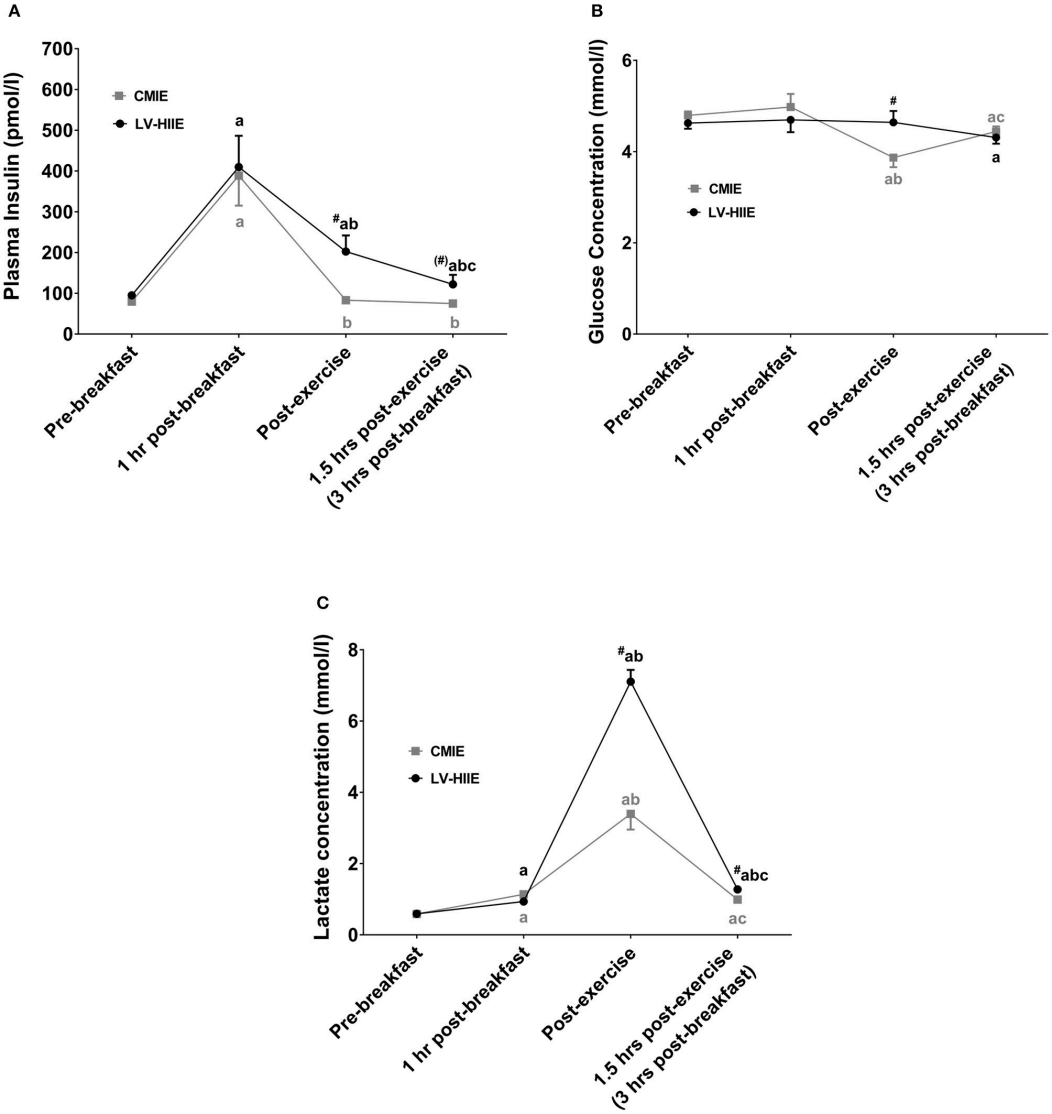
**Biochemical blood and plasma response to breakfast and exercise**. The postprandial and post-exercise response of plasma insulin **(A)**, blood glucose **(B)**, and blood lactate **(C)**. ^a^*p* < 0.05 compared to baseline; ^b^*p* < 0.05 compared to 1 h post-breakfast; ^c^*p* < 0.05 compared to post-exercise; ^#^*p* < 0.05 compared to the equivalent time point in the CMIE group. Symbols in parenthesis indicate *p* < 0.1. CMIE, continuous moderate intensity exercise; LV-HIIE, low-volume high-intensity interval exercise. Values are means ± SEM.

Blood glucose, plasma insulin, hydrogen peroxide, TBARS, catalase activity, and SOD activity were not significantly different between groups at baseline (all *p* > 0.05). Significant interaction effects were detected for plasma insulin (*p* = 0.01), glucose (*p* = 0.01), lactate (*p* < 0.01), hydrogen peroxide (*p* = 0.05), and TBARS (*p* < 0.01). *Post-hoc* analysis revealed that plasma insulin was significantly higher 1 h after breakfast compared to baseline for both the LV-HIIE and CMIE group (Figure 4). Immediately, and 1.5 h after CMIE, insulin levels returned to baseline. This was not evident after LV-HIIE where insulin levels remained significantly elevated. Blood glucose levels were significantly lower than baseline immediately after exercise with CMIE, and significantly lower than baseline 1.5 h after exercise after both CMIE and LV-HIIE (Figure 4). Blood lactate was significantly higher at all-time points after LV-HIIE compared to CMIE (Figure 4).

**Figure 5:**
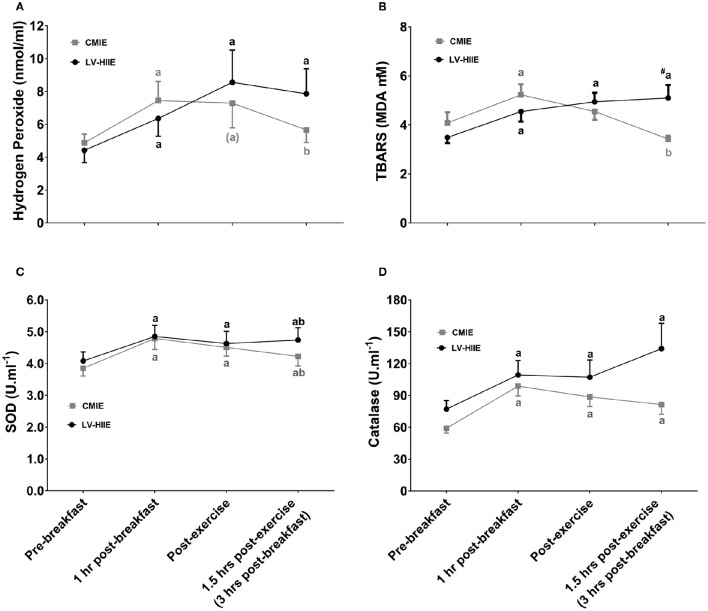
**Plasma redox status response to breakfast and exercise**. Plasma redox status response to ingestion of a standard breakfast and when either LV-HIIE or CMIE was performed 1 h after breakfast. The postprandial and post-exercise response of plasma hydrogen peroxide **(A)**, TBARS **(B)**, SOD activity **(C)**, and catalase activity **(D)**. ^a^*p* < 0.05 compared to baseline; ^b^*p* < 0.05 compared to 1 h post-breakfast; ^#^*p* < 0.05 compared to the equivalent time point in the CMIE group. Symbols in parenthesis indicate *p* < 0.1. CMIE, continuous moderate intensity exercise; LV-HIIE, low-volume high-intensity interval exercise; SOD, superoxide dismutase; TBARS, thiobarbituric acid reactive substances. Values are means ± SEM.

Plasma hydrogen peroxide, TBARS, catalase activity, and SOD activity were significantly higher 1 h after breakfast (Figure 5). SOD activity and catalase activity remained significantly elevated during the recovery period after both CMIE and LV-HIIE. On the other hand, *post-hoc* analysis of hydrogen peroxide and TBARS remained significantly elevated during the recovery period after LV-HIIE only (Figure 5).

### The effect of exercise on 3-h postprandial oxidative stress

A significant interaction (group*time*day) effect (*p* = 0.03) was detected for hydrogen peroxide. *Post-hoc* analysis indicated that compared to baseline hydrogen peroxide was significantly elevated 3 h after breakfast on the rest day with both CMIE and LV-HIIE groups (*p* < 0.05; Table 3). Furthermore, 3-h postprandial hydrogen peroxide was elevated to a greater extent (*p* < 0.05) on the exercise day compared to the rest day with LV-HIIE but not CMIE (Table 3). There was a trend toward significance (*p* = 0.06) for an interaction effect (group^*^time^*^day) for TBARS (Table 3). *Post-hoc* analysis indicated that compared to the rest day there was a tendency for greater TBARS at 3 h postprandial compared to the exercise day with LV-HIIE (*p* = 0.08) and lower TBARS on the exercise day with CMIE (*p* = 0.07). Furthermore, TBARS was significantly greater on the exercise day at 3 h postprandial with LV-HIIE compared to CMIE (*p* < 0.05; Table 3). A main time effect (*p* < 0.01) indicated significantly greater catalase activity at 3 h postprandial compared to baseline irrespective of exercise group or day (Table 3). A significant interaction (group^*^time^*^day; *p* = 0.043) effect was detected for SOD activity. *Post-hoc* analysis indicated significantly greater SOD activity at 3 h postprandial compared to baseline with both CMIE and LV-HIIE groups on the rest day, and with LV-HIIE on the exercise day (all *p* < 0.05). Compared to the rest day, there was a trend (*p* = 0.051) for decreased SOD activity at 3 h postprandial on the exercise day with CMIE.

**Table 3:** **The effect of exercise intensity and volume on plasma postprandial oxidative stress**.

	**Rest Day**			**Exercise Day**			
	**Baseline**	**3 h postprandial**	**Change on rest day (Cohens d)**	**Baseline**	**3 h postprandial**	**Change on exercise day (Cohens d)**	**Change between exercise and rest day (Cohens d)**
**HYDROGEN PEROXIDE (nmol.ml**^−1^**)**							
LV-HIIE	4.5 ± 0.8	5.6 ± 1.0^*^	1.1 ± 0.5 (0.37)	4.4 ± 0.7	7.9 ± 1.5^*^^†^	3.4 ± 1.3 (1.46)	2.3 ± 1.1^#^ (1.44)
CMIE	5.4 ± 0.8	6.8 ± 1.0^*^	1.4 ± 0.4 (0.46)	4.9 ± 0.5	5.7 ± 0.7	0.8 ± 0.6 (0.34)	−0.6 ± 0.4 (−0.36)
**TBARS (MDA μM)**							
LV-HIIE	3.2 ± 0.2	4.0 ± 0.3	0.8 ± 0.3 (0.68)	3.5 ± 0.2	5.1 ± 0.5^*^^#^^†^	1.6 ± 0.6 (1.30)	0.8 ± 0.8^#^ (0.52)
CMIE	4.1 ± 0.4	4.6 ± 0.4	0.5 ± 0.5 (0.44)	4.1 ± 0.4	3.4 ± 0.1^†^	−0.7 ± 0.4 (−0.53)	−1.2 ± 0.5 (−0.77)
**CATALASE (U.ml**^−1^**)**							
LV-HIIE	32.6 ± 2.4	42.9 ± 5.9^*^	10.3 ± 5.1 (1.24)	32.8 ± 3.3	57 ± 9.8^*^	24.2 ± 8.3 (2.25)	13.9 ± 8.4 (0.73)
CMIE	25.2 ± 1.6	39.2 ± 6.0^*^	14.0 ± 5.2 (1.69)	25.1 ± 1.9	34.6 ± 3.8^*^	9.5 ± 3.9 (0.88)	−4.5 ± 6.0 (−0.24)
**SOD (U.ml**^−1^**)**							
LV-HIIE	4.1 ± 0.3	4.5 ± 0.3^*^	0.4 ± 0.1 (0.44)	4.1 ± 0.3	4.7 ± 0.4^*^	0.7 ± 0.3 (0.69)	0.2 ± 0.2^#^ (0.50)
CMIE	3.9 ± 0.3	4.6 ± 0.3^*^	0.7 ± 0.1 (0.76)	3.9 ± 0.2	4.2 ± 0.3^†^	0.4 ± 0.2 (0.39)	−0.3 ± 0.2 (−0.68)

*Values are mean ± SEM. LV-HIIE, low-volume high-intensity interval exercise; CMIE, continuous moderate-intensity exercise; SOD, superoxide dismutase; TBARS, thiobarbituric acid reactive substances*;

* *p < 0.05 compared to baseline*;

† *p < 0.05 compared to rest day*;

# *p < 0.05 compared to CMIE; symbols in parenthesis indicate p < 0.1*.

Comparison of the change (3-h postprandial value minus the baseline value) between the rest day and exercise days indicated that LV-HIIE elicited a greater increase in hydrogen peroxide (*p* = 0.03), TBARS (*p* = 0.06), and SOD activity (*p* = 0.04) on the exercise day compared to CMIE.

## Discussion

We report the novel finding that LV-HIIE performed 1 h after consumption of a standard breakfast elicits a greater postprandial oxidative stress response compared to CMIE. Yet, over the 24-h post-exercise period LV-HIIE improves glycemic control to a similar extent as CMIE in overweight and obese adults. LV-HIIE consisted of significantly less work and time commitment compared to CMIE and therefore appears to be an effective exercise mode for incorporation into exercise programs designed to improve glycemic control in overweight and obese populations including insulin resistant conditions like PCOS.

### Acute exercise and 24-h glycemic control

Previous research indicates that a single bout of endurance exercise improves insulin sensitivity in the hours after exercise (Gillen et al., 2012; van Dijk et al., 2012; Little et al., 2014), however the effects of HIIE on glycemic control in sedentary overweight and obese populations are less clear. Laboratory based methods, such as the hyperinsulinaemic-euglycemic clamp and oral glucose tolerance test, may not reflect physiological insulin and glucose dynamics (Muniyappa et al., 2008) and can underestimate functional improvements in glycemic control (Mikus et al., 2012). We report the novel finding that 24-h post-exercise glycemic control measured under free living conditions is improved with LV-HIIE to a similar extent as CMIE. Manders et al. (2010) previously reported that CMIE (60 min at 35% W_max_) elicited greater improvements in 24-h glycemic control compared to higher-intensity exercise (30 min at 70% W_max_) in participants with type 2 diabetes. The discrepancy in findings may be a result of the different populations investigated, participants with type 2 diabetes vs. overweight/obese adults. However, Terada et al. (2016) recently reported greater improvements in 24-h glycemic control and postprandial glycemia after fasted-state HIIE (15 × 1 min at 100% VO_2peak_; 3-min active recovery periods) compared to work-matched CMIE treadmill exercise (60 min at 55% VO2_peak_) in participants with type 2 diabetes. Furthermore, HIIE cycling (10 × 1 min at 90% W_peak_; 1 min recovery periods at 15% W_peak_) in overweight/obese adults elicits greater improvements in 24-h glycemic control compared to work-matched CMIE (30 min at 35% W_peak_; Little et al., 2014). Combined with the current findings, HIIE is a potent stimulus for improving glycemic control, with potentially greater benefits occurring with HIIE of sufficient volume. In contrast, research exploring sprint-interval exercise reported no improvement in insulin sensitivity at 24 and 72 h post-exercise (Richards et al., 2010; Whyte et al., 2013), although insulin sensitivity was improved 24 h after an extended sprint work-matched to sprint-interval exercise (Whyte et al., 2013). However, these studies did not measure glycemic control during the post-exercise recovery period (Richards et al., 2010; Whyte et al., 2013). We extend previous findings by reporting that LV-HIIE, which consisted of substantially less time commitment and total work than CMIE, elicits similar improvements in 24-h post-exercise glycemic control.

Regulation of glycemic fluctuations and postprandial glycaemia are important for the long-term maintenance of insulin sensitivity and lowered risk of metabolic disease (Wright et al., 2006). Similar to previous reports (Little et al., 2014), we did not detect significant changes in glycemic variability as measured by MAGE, SD, and CV, with either LV-HIIE or CMIE. It is possible that differences were not detected, as CGM readings have been reported to underestimate measures of glycemic variability (Akintola et al., 2015). Furthermore, greater glycemic variability is strongly associated with a history of diabetes, suggesting that glycemic variability may play less of a role in apparently healthy populations who have regular glycemic control (Sartore et al., 2012). Nevertheless, the significant reductions in hyperglycemia, improved postprandial dinner response and improved 24-h average blood glucose and AUC, identify LV-HIIE as a beneficial exercise mode for improving overall glycemic control in sedentary, overweight and obese individuals. Indeed, training programs incorporating LV-HIIE are reported to promote long term improvements in glycemic control (Gibala et al., 2012). We extend these findings by reporting that a single session of LV-HIIE or CMIE can have similar improvements in glycemic control for up to 24 h after exercise has ceased.

### Redox status response to exercise performed 1 h after breakfast

Excessive oxidative stress leads to the development and progression of numerous pathologies including insulin resistance and type 2 diabetes (Valko et al., 2007; Fisher-Wellman and Neufer, 2012). Increased energy substrate availability following the consumption of a meal, results in elevated ROS production through mitochondrial membrane electron leak and the formation of AGEs (Tucker et al., 2008; Fisher-Wellman and Neufer, 2012). Excess ROS activate stress and mitogen activated protein kinase signaling pathways in insulin sensitive tissues contributing to the development of insulin resistance and type 2 diabetes (Wright et al., 2006; Tiganis, 2011). However, research has also highlighted exercise-induced ROS as a prominent moderator of glycemic control (Ristow et al., 2009; Trewin et al., 2015; Parker et al., 2016).

We report increased postprandial oxidative stress (TBARS and hydrogen peroxide) and antioxidant activity (catalase and SOD activity) 1 h after breakfast. In addition, hydrogen peroxide, catalase and SOD activity remain elevated 3 h after breakfast. Interestingly, only CMIE attenuated this postprandial oxidative stress response as evident by decreased TBARS and hydrogen peroxide 1.5 h after exercise compared to pre-exercise values, and decreased plasma TBARS 1.5 h after exercise compared to the rest day. Taken together with previous work (Canale et al., 2014), our findings suggest that postprandial oxidative stress is attenuated by CMIE, possibly due to improved clearance of plasma glucose. Certainly, others have reported that CMIE (1 h at 60% HR_max_) performed 2 h after a high fat meal attenuates postprandial oxidative stress in trained males (Mc Clean et al., 2007). In contrast, others have reported that cycling at 65–70% heart rate reserve for 45–60 min did not attenuate postprandial oxidative stress (Melton et al., 2009; Canale et al., 2014). The discrepancy in findings are likely related to the timing of meal ingestion, with improvements in postprandial oxidative stress occurring when exercise is performed in the hours after meal ingestion (Mc Clean et al., 2007), whereas exercising prior to meal ingestion may be less effective (Melton et al., 2009; Canale et al., 2014).

A novel finding was that plasma hydrogen peroxide and TBARS were elevated 1.5 h after LV-HIIE (approximately 3 h after breakfast) compared to the rest day. The mechanisms for the divergent oxidative stress response between LV-HIIE and CMIE are unclear. Oxidative stress is reported to be greater after higher-intensity exercise (Fisher-Wellman and Bloomer, 2009) and likely occurs through pathways independent of postprandial-induced oxidative stress (Fisher-Wellman and Bloomer, 2009; Fisher-Wellman and Neufer, 2012; Radak et al., 2013). Thus, it is likely that elevated oxidative stress after LV-HIIE is a result of increased exercise-induced oxidative stress. Additionally, it is possible that comparatively lower blood glucose after CMIE may allow for decreased mitochondrial electron leak, AGE formation, and subsequent ROS production (Wright et al., 2006; Tucker et al., 2008; Fisher-Wellman and Neufer, 2012).

Exercise-induced oxidative stress and subsequent redox-sensitive protein signaling facilitate many of the health benefits of acute and regular exercise (Ristow et al., 2009; Radak et al., 2013; Trewin et al., 2015; Parker et al., 2016). Furthermore, many of the metabolic health benefits of higher-intensity exercise occur during the delayed exercise recovery period (i.e., the day after exercise), potentially through alterations in redox status (Tyldum et al., 2009; Gabriel et al., 2012). It is possible that increased exercise-induced oxidative stress after LV-HIIE may be beneficial. Further research is required to elucidate the effect of LV-HIIE on redox status during the 24-h post-exercise recovery period.

### Glucoregulatory response to exercise performed 1 h after breakfast

Elevated postprandial glycemia is reported to play a role in the development of insulin resistance and metabolic disease (Wright et al., 2006). Similar to previous research (van Dijk et al., 2013), we demonstrate that a single session of CMIE performed 1 h after breakfast attenuates postprandial glycemia. We extend previous findings by indicating that LV-HIIE also attenuates postprandial glycemia, despite consisting of considerably less total work and time-commitment. CMIE decreased whole blood glucose immediately after exercise compared to baseline and LV-HIIE. This reduction was transient as both LV-HIIE and CMIE elicited a similar decrease in whole blood and CGM glucose measures 1.5 h after exercise. Circulating plasma catecholamines are reported to increase during high-intensity exercise which leads to a 7–8-fold increase in hepatic glucose production compared to moderate intensity exercise (Marliss and Vranic, 2002). During recovery, catecholamine concentrations rapidly decrease, removing the catecholamine inhibition of glucose-stimulated insulin secretion (Marliss and Vranic, 2002). This leads to elevated plasma glucose and insulin levels after high-intensity exercise compared to moderate intensity exercise. This stress hormonal response may explain the comparatively higher glucose levels immediately after LV-HIIE, and higher post-exercise insulin levels throughout the recovery period, compared to CMIE. It is important to note that this elevated glucose response with LV-HIIE was transient and did not negatively impact the 24-h improvements in glycemic control post-exercise.

### Limitations and strengths

A strength of this study was the recruitment of inactive and overweight and obese men and women including a subgroup of women with PCOS. Although, sex differences in glycemic control (Gillen et al., 2014) and postprandial oxidative stress have been reported (Bloomer and Lee, 2013), sample size and participant characteristics were well matched between LV-HIIE and CMIE exercise groups, and all females were tested in the early follicular phase of the menstrual cycle. A small sample size prevented subgroup analysis to explore whether particular groups (men, women, and/or women with PCOS) had different glycemic control responses to the exercise sessions. Despite this limitation, this study provides preliminary data to suggest that overweight and obese men, women, and women with PCOS, may be able to improve 24-h glycemic control with a single session of CMIE or LV-HIIE. Future research is warranted to investigate glycemic responses in these subgroups. Plasma TBARS (malondialdehyde) and hydrogen peroxide have previously been used to reflect systemic postprandial oxidative stress (Bloomer et al., 2010; Bloomer and Lee, 2013; Canale et al., 2014). Nevertheless, future research would benefit from employing additional measures of oxidative stress in plasma such as the oxidized/reduced glutathione ratio or the direct measurement of ROS through spin trapping and electron spin resonance spectroscopy. Another potential study limitation is that the participants were not blinded to real-time CGM readings, which when prompted are displayed on an LCD screen. If possible, future studies should adopt a CGM system such as the iPro®2 Professional CGM (Medtronic MiniMed, Northridge, CA) which allows blinding of participants to real-time CGM readings. A potential limitation of the study is the natural variability of outcome measures between the two exercise groups which is inherent to a parallel design study. Despite appropriate matching of participant characteristics and the inclusion of the rest-control day for exercise day comparisons, future research would benefit from adopting a cross-over design study to confirm these findings. In summary, further cross-over randomized control trials are warranted to explore the acute and chronic impacts of LV-HIIE in larger groups of people in specific clinical populations, and using more diverse exercise protocols including that of work-matched CMIE.

## Conclusions

A single session of CMIE, but not LV-HIIE, attenuated postprandial plasma hydrogen peroxide and glycemia when performed 1 h after breakfast consumption. Yet, over the 24-h post-exercise period LV-HIIE elicited similar improvements in glycemic control to CMIE. Given its time-efficient nature, LV-HIIE may be an effective exercise mode to incorporate into exercise programs for the improvement of 24-h glycemic control in inactive, overweight and obese adults. Furthermore, measuring glycemic control immediately after exercise may not accurately reflect functional improvements over the 24-h post-exercise recovery period, especially with respect to high-intensity exercise.

## Author contributions

LP, CS, LB, IL, KH, AM, and NS contributed to the study design and acquirement of ethical approval. LP, CS, LB, and NS contributed to data collection. LP analyzed the data and drafted the initial manuscript. The remaining authors critically revised the manuscript. All authors approved the final version of the manuscript. NS, LP are guarantors of the manuscript and take full responsibility for the work as a whole, including the study design, access to data, and the decision to submit and publish the manuscript.

## Funding

This work is supported by the Australian Government Collaborative Research Network (CRN) awarded to the authors LP, CS, IL, AM, and NS.

### Conflict of interest statement

The authors declare that the research was conducted in the absence of any commercial or financial relationships that could be construed as a potential conflict of interest.
